# Presurgical olfactory function as an indicator of the outcome of functional endoscopic sinus surgery in chronic rhinosinusitis with nasal polyps

**DOI:** 10.1007/s00405-022-07496-3

**Published:** 2022-06-24

**Authors:** Constantin A. Hintschich, Jürgen Pade, Petros Petridis, Thomas Hummel

**Affiliations:** 1 Department of Otorhinolaryngology, Regensburg University Hospital, Franz-Josef-Strauss-Allee 11, 93053 Regensburg, Germany; 2 Department of Otorhinolaryngology, St. Johannes Municipal Hospital, Dortmund, Germany; 3 Smell and Taste Clinic, Department of Otorhinolaryngology, TU Dresden, Dresden, Germany

**Keywords:** Chronic rhinosinusitis, Olfaction, FESS, Smell

## Abstract

**Introduction:**

Chronic rhinosinusitis with nasal polyps (CRSwNP) often leads to impaired olfactory function and reduced quality of life. When conservative treatments such as nasal irrigation and topical steroids fail, functional endoscopic sinus surgery (FESS) is often necessary, because it improves symptoms and enhances quality of life.

**Materials and methods:**

A total of 88 patients was included in this prospective study. All subjects underwent an extensive examination both presurgically and 4 months after operations including nasal endoscopy and psychophysical olfactory testing (Sniffin’ Sticks). Moreover, disease-specific quality of life was assessed and presurgical CT scans were rated regarding the opacification of the paranasal sinuses.

**Results:**

Presurgically psychophysical tests showed an overall olfactory dysfunction. Olfactory test results (TDI score) correlated with endoscopic (Lund–Kennedy and Lildtholdt score) and CT scores (Lund–Mackay and TOCS scores). Four months after surgery olfactory function was enhanced and quality of life significantly showed an overall improvement. However, the outcome was dependent on the extent of presurgical olfactory function: olfaction and quality of life improved most pronounced in anosmics compared to hyposmic and especially normosmic patients.

**Conclusions:**

This study confirmed that FESS in CRSwNP leads to a significant improvement of both olfaction and disease-specific quality of life. Moreover, preoperative psychophysical assessment of the extent of olfactory dysfunction can help to objectively assess possible risks and expected benefits of the surgery in terms of olfaction and quality of life.

## Introduction

Chronic rhinosinusitis (CRS) which can be subdivided into a subtype with nasal polyps (CRSwNP) and one without nasal polyps (CRSsNP) is a widely underestimated medical condition. Its prevalence among the European population has been shown to be between 6.9 and 27.1% [1].

Besides nasal discharge, congestion and facial pain/pressure, olfactory dysfunction is a frequent symptom. Smell impairment occurs in up to 78% [2, 3]. In reverse, CRS is the most common etiology for olfactory dysfunction [4]. This is due to two different pathomechanisms: First, mucosal swelling, with polyps or secretions blocking the olfactory cleft resulting in a conductive olfactory loss. Hence, olfactory molecules cannot access the olfactory mucosa [5, 6]. Second, inflammatory cytokines such as TNF-alpha mediate the invasion of inflammatory cells into the olfactory epithelium. The inflammation leads to an erosion of the olfactory neuroepithelium and sensorineural olfactory loss [6–8], and also to functional impairment of the olfactory receptor neurons.

The first-line therapy of CRS is nasal irrigation and topical steroids, leading in many cases to a marked improvement of the symptoms including olfaction [9]. If these conservative measures fail, functional endoscopic sinus surgery (FESS) is often indicated. Especially, in CRSwNP, surgery significantly improves specific symptoms as well as quality of life [10]. In addition, olfaction recovers both in subjective and psychophysical measures [11–14].

The aim of this study was (1) to assess olfactory outcome and quality of life after FESS in CRSwNP and (2) to identify possible prognostic factors on both postoperative olfaction and quality of life. (3) In addition, we examined correlations between olfactory function and both CT and endoscopic scores.

## Methods

A total of 154 patients participated in this study. Between Mai 2018 and August 2019, these patients underwent FESS for CRSwNP at the Department of Otorhinolaryngology, St. Johannes Municipal Hospital, Dortmund, Germany. The extent of the operations was based on the pathology and, however, includes in all cases infundibulotomy and ethmoidectomy. All surgeries were performed by the same surgeon (TP). The day before surgery all patients had an endoscopic examination and psychophysical olfactory testing. In addition, they completed a questionnaire regarding the patient’s demographics, CRS-related conditions, a self-evaluation of the sense of smell as well as the German adapted version of the Sino-nasal Outcome Test (SNOT-20) [15]. Paranasal CT imaging was evaluated regarding the opacification of the olfactory cleft.

Four months postoperatively, all patients were invited to a follow-up; 88 patients attended this visit and were evaluated by the same extensive examinations and questionnaires as preoperatively.

### Psychophysical olfactory testing

Psychophysical testing was performed both 1 day before surgery and in the follow-up using the Sniffin’ Sticks smell test (Burghart Messtechnik, Germany) [16]. This blinded and validated test allows a specific evaluation of phenyl ethylalcohol odor threshold, odor identification, and odor discrimination. The corresponding subscores for threshold (T, maximum 16 points), identification (I, maximum 16 points) and discrimination (D, maximum 16 points) sum up to the composite TDI score (maximum 48 points). Pre-established cutoff values were used for functional anosmia (≤ 16), hyposmia (16.25—30.5), and normosmia (> 30.5) [17].

### Endoscopic evaluation

Both preoperatively and in the follow-up, an endonasal endoscopy was performed and evaluated using both the Lildholdt score and Lund–Kennedy score The evaluation of the size of polyps (0: no polyps; 1: polyps not reaching the inferior turbinate; 2 = polyps not reaching the lower edge of the inferior turbinate; 3 = polyps reaching below the lower edge of the inferior turbinate) was done for each side separately and then summed up to the Lildholdt score [18]. The presence and severity of potential polyps, edema, discharge, scarring, and crusting were assessed with scores between 0 and 2, for both sides separately. All subscores were then summed up to the Lund–Kennedy score [19].

### Evaluation of the CT

The preoperative paranasal CT scans were graded accordingly to the Lund–Mackay score and the total opacification of the olfactory cleft (TOCS): For the Lund–Mackay score, each ipsilateral sinus system (anterior and posterior ethmoid cells separately) and the osteomeatal complex has been scored 0 (no abnormality), 1 (partial opacification), or 2 (total opacification). The final score (maximum 24) is the sum of the single values [20].

The opacification of the olfactory cleft has been evaluated using the TOCS according to Chang et al. [21] and the later modification by Kim et al. [22]: for the anterior and posterior olfactory cleft of each side, the opacification was ranked between 0 (no opacification) and 4 (total opacification). The subscores of the anterior and posterior olfactory cleft of each side were added up to TOCS (maximum score: 16).

### Assessment of quality of life

The disease-specific, health-related quality of life was assessed by the validated German-adapted version of the SNOT-20 [15]. This questionnaire consists of 20 questions and is a well-established questionnaire to quantify sinunasal symptoms and to evaluate the treatment outcome in CRS in 20 questions [23].

### Statistical analyses

Data were analyzed using SPSS Statistics software (version 26, IBM, Armonk, NY, USA). Graphs were illustrated using Prism software (version 9, GraphPad Software, San Diego, CA, USA). Values are expressed as mean ± standard deviation (SD), and *p* < 0.05 was considered statistically significant. Continuous data were tested for statistical significance using unpaired two-tailed Student’s *t* tests or one-way ANOVA. Pearson’s correlation was used to assess the correlation between the scores.

## Results

### Patients’ demographics

Of 154 preoperatively assessed patients, a total of 88 patients (51 ± 14 years) attended the follow-up visit 120 ± 49 days after surgery and were, therefore, included in the study. 37 patients were female (42%), and 51 patients were male (58%). 36 patients (41%) underwent a revision surgery. Presurgically, 67 and 2 patients were treated with topical and systemic steroids, respectively. The patients’ demographics and CRS-related comorbidities are displayed in Table 1.

**Table 1:** Demographics, CRS-related comorbidities, results of olfactory testing, quality of life questionnaire, radiological and endoscopic scores of the entire cohort and the subgroups

	All (*n* = 88)	Anosmic (*n* = 44)	Hyposmic (*n* = 34)	Normosmic (*n* = 10)	
Age (years)	51 ± 14	52 ± 12	53 ± 15	44 ± 17	
Female	42%	50%	32%	40%	
Interval (days)	120 ± 49	121 ± 42	122 ± 59	115 ± 37	
BMI (kg/m^2^)	27.3 ± 4.9	26.5 ± 4.0	28.4 ± 5.9	26.8 ± 4.4	
Aspirin sensitivity	10%	14%	3%	20%	
Asthma	29%	45%	12%	20%	
Tissue eosinophilia	71%	88%	50%	67%	

	Preoperative	Follow-up	Preoperative	Follow-up	Preoperative	Follow-up	Preoperative	Follow-up
TDI	17.3 ± 10.1	22.7 ± 8.5	8.4 ± 3.9	18.0 ± 8.2	24.0 ± 4.4	26.3 ± 6.0	33.3 ± 1.4	31.2 ± 4.1
Threshold	2.0 ± 2.5	3.4 ± 3.0	0.2 ± 0.4	1.9 ± 1.9	3.2 ± 2.4	4.4 ± 3.2	5.7 ± 1.5	6.4 ± 2.5
Discrimination	7.8 ± 4.2	9.5 ± 3.5	4.4 ± 2.0	8.3 ± 3.6	10.4 ± 2.4	10.4 ± 3.0	14.1 ± 1.2	11.5 ± 2.5
Intensity	7.5 ± 4.4	9.8 ± 3.7	3.8 ± 2.4	7.8 ± 3.8	10.4 ± 2.2	11.5 ± 2.2	13.5 ± 1.0	13.3 ± 1.3
SNOT-20	38.5 ± 16.0	20.0 ± 13.5	43.1 ± 15.8	22.4 ± 13.5	35.1 ± 15.6	16.7 ± 12.9	29.9 ± 13.1	22.0 ± 14.4
Primary nasal symptoms	56.2 ± 18.3	26.2 ± 17.3	63.8 ± 15.3	30.6 ± 17.6	49.2 ± 18.8	20.7 ± 15.9	46.4 ± 15.3	26.0 ± 16.4
Secondary rhinogenous symptoms	31.1 ± 18.8	17.6 ± 14.6	35.2 ± 18.5	21.2 ± 16.0	28.6 ± 18.6	13.3 ± 12.6	22.0 ± 17.9	16.3 ± 10.6
General quality of life	33.7 ± 19.8	18.5 ± 15.8	37.0 ± 20.3	18.7 ± 15.0	31.7 ± 18.8	16.7 ± 16.1	26.0 ± 19.6	23.6 ± 18.7
CT scores								
Lund–Mackay score	14.2 ± 6.5		17.8 ± 4.3		11.9 ± 6.1		6.9 ± 5.9	
TOCS	9.4 ± 5.3		13.3 ± 2.9		6.4 ± 4.4		3.0 ± 2.4	
Endoscopic scores								
Lund–Kennedy score	7.7 ± 5.0	5.7 ± 4.1	9.6 ± 6.1	6.6 ± 5.0	6.1 ± 2.6	4.9 ± 2.6	4.9 ± 2.4	3.4 ± 2.1
Lildholdt score	3.1 ± 1.3	0.6 ± 0.9	3.4 ± 1.3	0.8 ± 1.0	2.8 ± 1.2	0.4 ± 0.8	2.4 ± 1.4	0.3 ± 0.5

### Self-evaluation of olfactory function and psychophysical smell test before surgery

Before surgery patients, self-rated their olfactory function as 3.6 ± 2.5 on a visual analogue score (VAS; 0: no olfactory function, 10 very good olfactory function). Psychophysical testing of olfactory function revealed a mean TDI of 17.3 ± 10.1. When applying the well-established boundaries for the TDI [17], the group could be categorized in 44 anosmic (50%), 34 hyposmic (39%) and 10 normosmic patients (11%). Interestingly, presurgical steroid medication or previous surgery did have no statistically significant impact on the preoperative TDI score (16.7 ± 9.8 vs. 19.4 ± 11.0, *p* > 0.05; 14.8 ± 9.5 vs. 19.0 ± 10.2, *p* > 0.05).

### Self-evaluation of olfactory function and psychophysical smell test after surgery

Following surgery olfactory function improved significantly better in both self-evaluation and psychophysical testing: Patients evaluated their subjective smell function as 6.0 ± 2.8 on the VAS. In the Sniffin’ Sticks test the cohort scored a mean TDI of 22.7 ± 8.5.

**Fig. 1:**
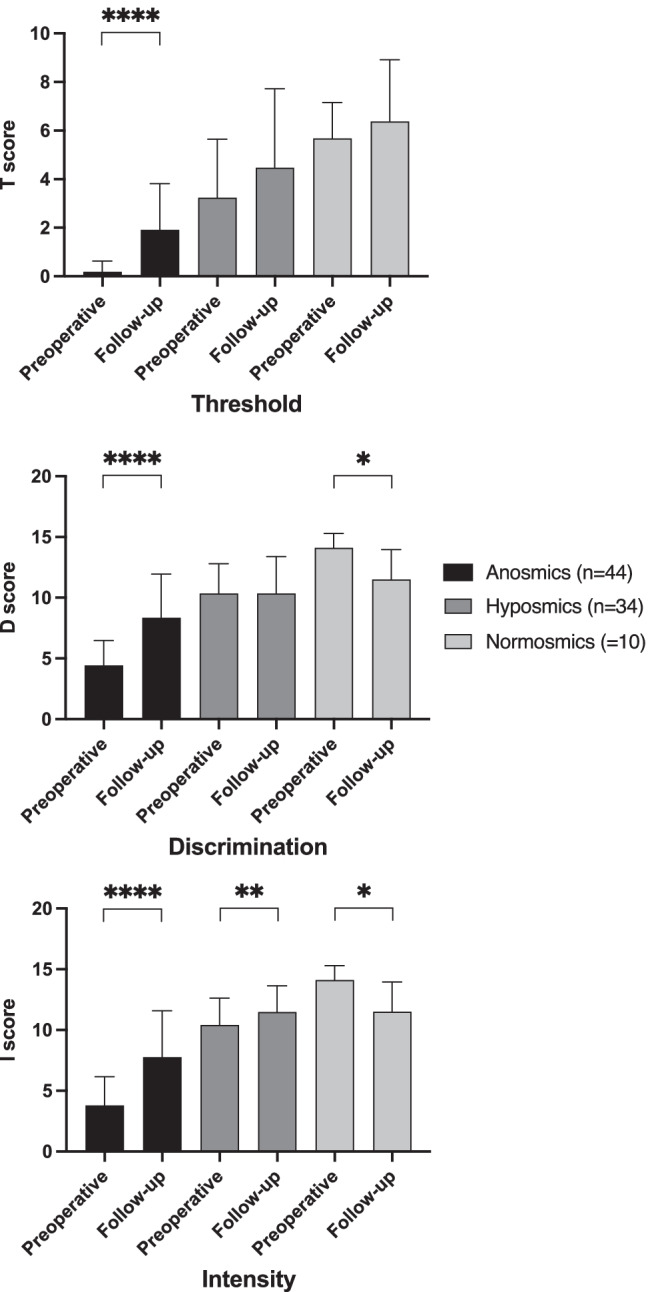
Box plots comparing the TDI subscores (* < 0.05; ** < 0.01; **** < 0.0001)

When the minimal clinically important difference of 5.5 points (MCID) was taken into account [24] olfactory function improved in 39 out of 88 patients (44%), did not change in 43 patients (49%) and deteriorated in 6 patients (7%). A (clinically important) positive effect of surgery on olfactory function was highest in previously anosmic (71%) compared to hyposmic (24%) and normosmic patients (0%). Vice versa, three hyposmic (9%) and three normosmic patients (30%) scored lower in the follow-up compared to the presurgical smell test. In the follow-up psychophysical testing revealed 20 anosmic (23%), 53 hyposmic (60%), and 15 normosmic patients (17%). TDI subscores are displayed in Fig. 1.

For both the presurgical and the follow-up examination patients’ subjective self-evaluation correlated strongly with the results of the Sniffin’ Sticks test (presurgically *r* = 0.69, *p* < 0.0001; follow-up *r* = 0.69, *p* < 0.0001).

### Preoperative paranasal CT

Presurgically, the TOCS, a radiological score for the opacification of the olfactory cleft, was 9.4 ± 5.3 over all patients. As expected, this score differed significantly between the three subgroups normosmia (3.0 ± 2.4), hyposmia (6.4 ± 4.4) and anosmia (13.3 ± 2.9; *p* < 0.001). Correspondingly, the TOCS had a strong negative correlation with the preoperative TDI (*r* = − 0.79, *p* < 0.001).

Overall patients, the Lund–Mackay score which describes the opacification of paranasal sinuses in the CTs, was 14.2 ± 6.5. As expected, this score differed significantly between the three subgroups normosmia (6.9 ± 5.9), hyposmia (11.9 ± 6.1) and anosmia (17.8 ± 4.3; *p* < 0.001). Likewise, the Lund–Mackay score had a moderate negative correlation with the preoperative TDI (r = -0.55; *p* < 0.001).

### Nasal endoscopy

Preoperative endoscopy revealed a Lund–Kennedy score and a Lindholdt score of 7.8 ± 5.1 and 3.1 ± 1.3, respectively. Both scores improved after the operation (5.7 ± 4.1; 0.6 ± 0.9; *p* < 0.001). In anosmic patients, both scores were higher (9.6 ± 6.1; 3.4 ± 1.3) than in patients with hyposmia (6.1 ± 2.6; 2.8 ± 1.2) and normosmia (4.9 ± 2.4; 2.4 ± 1.4).

Both preoperatively and in the follow-up, these two endoscopic scores correlated negatively with TDI, too (*r* = − 0.45, *p* < 0.001 and *r* = − 0.47, *p* < 0.001 for Lund–Kennedy score, *r* = − 0.35, *p* < 0.001 and *r* = − 0.35, *p* < 0.001 for Lindholdt score).

### Disease-specific quality of life

FESS improved overall SNOT-20 score most significantly (ΔSNOT-20: 18.4 ± 15.5; *p* < 0.001). When applying the MCID of 16 [23], a total of 52 patients (59%) benefitted and no patient worsened from the operation. However, again, there was a substantial difference between the subgroups: Patients with presurgical anosmia and hyposmia improved in 64% and 62%, respectively. However, only 30% of normosmic patients experienced a MCID in the SNOT-20.

Interestingly, no significant correlation was seen between the change in SNOT-20 and the change in TDI (*r* = − 0.17, *p* = 0.11).

## Discussion

In this prospective study, we assessed olfactory function, endonasal endoscopy, opacification of the paranasal CT and disease-specific quality of life before FESS and reevaluated all but radiological measures 4 months after surgery.

Over all patients, FESS led to an enhanced olfaction in both self-evaluation and psychophysical testing and hence confirmed previous data [11, 12, 25]. When applying the MCID psychophysically assessed olfaction improved in 44% of the patients. The TDI score did not change clinically important in 49% and even decreased in 7% following surgery. Despite its overall positive effects on the sense of smell, the risk of a postsurgical impairment of olfaction is well-known after FESS and has been previously described between 2 and 9% [12, 25, 26]. It may be that in a few cases, the inflammatory process is activated following the surgical procedures.

However, when the population was split up according to their TDI score into anosmic patients (*n* = 44), hyposmic (*n* = 34) and normosmic patients (*n* = 10), the olfactory outcome differed considerably between these groups: 70% of anosmic and 24% of hyposmic patients improved in terms of psychophysically assessed olfaction. Contrary, in normosmic patients, no clinically important improvement of olfaction could be seen four months after surgery. On the other hand, 9% of hyposmic and even 30% (n = 3) of normosmic patients experienced smell loss on a clinically important level.

Hence, surgery improved olfaction overall and especially in patients with an impaired olfaction. However, postsurgically only the minority of patients were normosmic. This is most likely not only due to possible iatrogenic injuries [27], but mainly due to a persisting sensorineural olfactory loss caused by the chronic inflammatory processes [6]. Consequently, to enhance olfactory outcome surgery should be considered already in an early stage when CRSwNP is refractory to conservative treatment and already associated with an olfactory impairment [28].

As shown previously, FESS in CRSwNP also had a positive effect on the disease-specific, health-related quality of life [29]. When a MCID was considered, 59% of all patients improved. This is in good accordance with previous publications, which showed an improved SNOT-20 (or the slightly modified version SNOT-22) after FESS in CRSwNP [30–32].

Again, the more impaired the presurgical olfaction, the more likely was the improvement of SNOT-20 after surgery: 64% of anosmic and 62% of hyposmic patients, but only 30% of normosmic patients experienced an improvement in the SNOT-20 (Fig. 1).

The Lund–Mackay score which is a measure for the opacification of all paranasal sinuses correlated moderately negatively with the preoperative TDI score, as published previously [12]. However, the correlation was even stronger between preoperative TDI score and TOCS which quantifies solely the opacification of the olfactory cleft. This has been described before in a similar but weaker correlation [22, 33].

The endoscopic Lund–Kennedy score and Lildholdt score correlated moderately and weakly, respectively, negatively with presurgical TDI score. A correlation between Lund–Kennedy score and preoperative olfaction is known for the Smell Intensity Test [3].

As in any surgical speciality the postoperative outcome is not only depended on technical aspects of the surgery itself, but essentially on the correct indication. In FESS in CRSwNP the improvement of symptoms and the quality of life is the principal aim and subsequently the basis to assess postoperative outcome. For CRSwNP up to now no prognostic scoring system has been established to evaluate the postsurgical outcome after FESS. Hence the indication on whether and when to operate is mostly dependent of the severity of the symptoms. As the surgeon could greatly benefit from a reliable criterion to realistically assess both benefits and risks of FESS in CRSwNP we assessed different preoperative measures as potential indicators for the surgical success.

Even if olfactory dysfunction in CRS is associated with a reduced quality of live [34], change of SNOT-20 did not correlate with change in TDI. Hence, the changes in both olfaction and quality of life can be seen as independent measures of the surgical outcome. Therefore, we compared potential indicators for the success of FESS measured in changed olfaction and quality of life. Different to a recent publication we did not see any correlation between endoscopic Lildholdt score and the change in TDI [26]. This might be due to a different patient distribution with a milder degree of polyposis in the previous study of Haxel et al. Interestingly, in our study only preoperative TDI but no radiological or endoscopic score correlated with both ΔTDI and ΔSNOT-20 (Table 2).

**Table 2:** Correlations between preoperative scores and the change in the TDI score and SNOT-20, respectively (n.s.: not significant)

	ΔTDI	ΔSNOT-20
TDI score	*r* = − 0.57, *p* < 0.001	*r* = 0.23, *p* = 0.032
CT scores		
Lund–Mackay score	*r* = 0.25, *p* = 0.021	n.s.
TOCS	*r* = 0.40, *p* < 0.001	n.s.
Endoscopic scores		
Lund–Kennedy score	n.s.	n.s.
Lildholdt score	n.s.	r = -0.26, *p* = 0.015

**Fig. 2:**
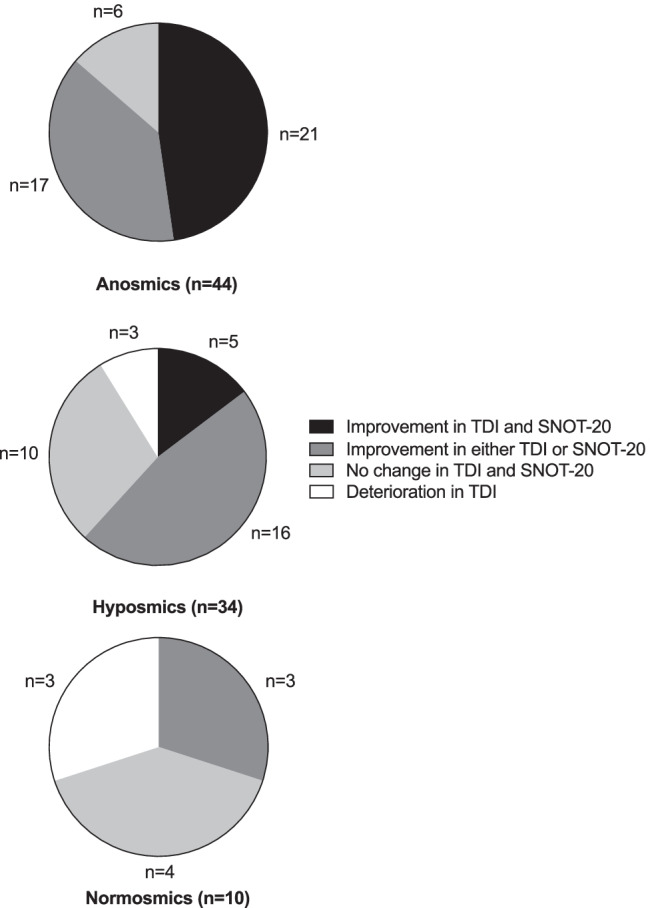
Pie charts for the change in TDI score and SNOT-20 as measure for of postsurgical outcome dependent on presurgical degree of olfactory impairment. Note that no deterioration in SNOT-20 has been observed

Therefore, classification of patients into anosmics, hyposmics, and normosmics appears to be a good indicator for the postsurgical outcome. In the anosmic group 48% of the patients benefitted of a (clinically important) improvement in both TDI score and SNOT-20. This was substantially higher than in hyposmic (15%) and normosmic patients (0%) (Fig. 2). Hence preoperative assessment of olfaction might be a valuable prognostic factor to estimate surgical outcome after FESS in CRSwNP.

However, there are some limitations to our study: First, a substantial portion of preoperatively included patients could not be assessed in the follow-up. This might cause a selection bias of the follow-up. Second, also the choice of the QoL-questionnaire could have influenced the results: In contrast to the later SNOT-22 the SNOT-20 does not include any questions on the chemical senses. This could explain the missing correlation between the change in SNOT-20 and the change in TDI. Future studies should address these limitations and could potentially also include CRSsNP as a control group.

## Conclusions

We confirmed previous studies, which showed an improvement of olfaction and quality of life through FESS in CRSwNP. Moreover, we could establish that the extent of preoperative olfactory dysfunction is an indicator for postsurgical success. Hence, preoperative psychophysical olfactory testing and classification into normosmia, hyposmia, and anosmia can help to objectively assess possible risks and expected benefits of the surgery in terms of quality of life and olfaction.
